# Sunflower as a ‘*Candidatus* Phytoplasma solani’ reservoir and crop-associated planthopper vectors (Cixiidae) sustain stolbur diseases in agroecosystems

**DOI:** 10.3389/fpls.2026.1859389

**Published:** 2026-06-22

**Authors:** Živko Ćurčić, Aleksandra Delić, Yann Galein, Werner Holzinger, Andrea Kosovac

**Affiliations:** 1 Section for Sugar Beet, Sunflower Department, Institute of Field and Vegetable Crops, Novi Sad, Serbia; 2 Florimond Desprez Belgium Group, Tienen, Belgium; 3 Oekoteam, Institute for Animal Ecology and Landscape Planning, Graz, Austria; 4 Laboratory of Applied Entomology, Institute of Pesticides and Environmental Protection, Belgrade, Serbia

**Keywords:** Fulgoromorpha, *Reptalus quinquecostatus*, *Reptalus panzeri*, stolbur phytoplasma, sunflower, maize, sugar beet, crop rotation

## Abstract

Understanding plant-vector-pathogen interactions is essential for explaining the persistence of stolbur diseases associated with the plant pathogenic bacterium ‘*Candidatus* Phytoplasma solani’ (CaPsol) in agroecosystems. This study investigates the role of crop-associated *Reptalus* planthoppers (Insecta: Hemiptera: Cixiidae) in shaping CaPsol epidemiology within a four-crop rotation system (sunflower, wheat, sugar beet, and maize) in Serbia under field and experimental conditions. Over a three-year period (2023–2025), nymphal surveys, adult monitoring, emergence-cage experiments, molecular identification of planthoppers, assessment of CaPsol in crops, and vector transmission assays were conducted, with a particular focus on the sunflower-wheat sequence. Sunflower was identified as a developmental host for *Reptalus quinquecostatus* and *Reptalus panzeri*, with high CaPsol infection rates detected in both the host plant and associated vectors. In contrast, wheat appeared to primarily support nymphal overwintering, while its significance as a CaPsol reservoir remains inconclusive. Furthermore, the maize-wheat sequence supported the development of *R. quinquecostatus* while confirming its role in the life cycle of *R. panzeri*. Experimental trials demonstrated efficient CaPsol transmission to sunflower but limited transmission to wheat. Despite high infection levels under field conditions, sunflower remained asymptomatic, suggesting its role as a cryptic pathogen reservoir. The findings indicate that the sunflower-wheat sequence may sustain CaPsol-infected *Reptalus* spp. populations across seasons, facilitating pathogen persistence within cropping systems. The study highlights the importance of integrating vector life cycle dynamics into epidemiological frameworks of stolbur diseases, particularly in crop rotations where major cultivated hosts may support in-field CaPsol pathosystems.

## 1 Introduction

Planthoppers (Hemiptera: Auchenorrhyncha: Fulgoromorpha) of the family Cixiidae are the principal insect vectors of the phloem-limited bacterium ‘*Candidatus* Phytoplasma solani’ (CaPsol; 16SrXII-A subgroup), the etiological agent of stolbur diseases (Quaglino et al., 2013; Jović et al., 2019). Since their first reports in Europe in the mid-20th century (Suhov and Vovk, 1949), these diseases have caused substantial agroeconomic losses, ranging from reduced yields to complete crop failure (Carraro et al., 2008; Navrátil et al., 2009; Ćurčić et al., 2021a). However, the mechanisms driving disease outbreaks remain insufficiently understood, largely due to complex multitrophic interactions among vectors, host plants, and the pathogen within agroecosystems. The host plant spectrum and epidemiology of CaPsol are are strongly influenced by vector ecology and life history, highlighting the need to move beyond a plant-centered perspective toward a broader, entomology-integrated approach (Sforza et al., 1998; Gatineau et al., 2001; Langer and Maixner, 2004; Chuche et al., 2016; Jović et al., 2019). More than five decades of research in Serbia have linked several planthopper species to stolbur diseases in multiple crops, providing an important regional framework for understanding CaPsol pathosystem in the central Balkans (Aleksić et al., 1967; Jović et al., 2009; Cvrković et al., 2014; Mitrović et al., 2016; Kosovac et al., 2019, 2023a, 2023b).

Although not fully elucidated, planthopper life cycles are generally characterized by subterranean nymphal development followed by a shorter adult flight period, with both stages capable of harboring and transmitting the pathogen (Nickel, 2003; Jović et al., 2009; Kaul et al., 2009). In the classical weed-associated pathosystem, the planthopper *Hyalesthes obsoletus* (Cixiidae) maintains CaPsol cycles through *Convolvulus arvensis* and *Urtica dioica*, which function as both vector host plants and pathogen reservoirs, making weed management central to disease control (Kehrli and Delabays, 2012; Mori et al., 2014). Increasing evidence, however, suggests that this model does not fully explain disease dynamics in intensively managed agroecosystems, where cultivated plants themselves may contribute to pathogen persistence. In this context, cixiid planthoppers of the genus *Reptalus* have emerged as important CaPsol vectors in several cultivated crops. *Reptalus cuspidatus* was only recently confirmed as a CaPsol vector to sugar beet, although its epidemiological significance and life history remain still largely unknown. Adults have been recorded on several herbaceous hosts, including *C. arvensis* and *Artemisia vulgaris*, both recognized CaPsol reservoirs (Picciau et al., 2008; Trivellone et al., 2016; Kosovac et al., 2023a). In maize-wheat crop sequence, *Reptalus panzeri* completes its development through a continuous cycle in which maize serves as the primary host and pathogen reservoir, while wheat supports overwintering and maintenance of infected vector populations (Jović et al., 2009). This finding demonstrates that cultivated plants can sustain CaPsol epidemiological cycles and serve as pathogen reservoirs, thereby intensifying seasonal disease pressure.

A congeneric planthopper, long referred to as *Reptalus quinquecostatus* (Dufour, 1833), has been recorded in vineyards and a range of crops as a suspected CaPsol vector (Trivellone et al., 2005; Jović et al., 2009; Cvrković et al., 2014; Mitrović et al., 2016; Chuche et al., 2016; Pierro et al., 2020). In a recent taxonomic revision, Webb et al. (2013) erroneously synonymized this name with *Reptalus panzeri* sensu Le Quesne, 1960 nec Löw, 1883 resp. *Reptalus melanochaetus* (Fieber, 1876). This led to further confusion: Emeljanov (2020) came to the conclusion that the valid name of the species previously named *R. quinquecostatus* is in fact *Reptalus artemisiae* (Becker, 1865), and four years later Tishechkin and Emeljanov (2024) assumed that the species did not yet have a validly published name and therefore introduced a new name for this taxon, *Reptalus salicinus* Tishechkin and Emeljanov, 2024. These conflicting interpretations have resulted in inconsistent nomenclature in studies addressing the CaPsol epidemiological role of this planthopper across different phloem-restricted pathosystems (e.g., Pfitzer et al., 2022; Pierro et al., 2022; Kreitzer et al., 2025). As we consider both *R. artemisiae* and *R. salicinus* as distinct species, not conspecific with the taxon from Serbia that has been experimentally confirmed as a CaPsol vector (Kosovac et al., 2023a, b), we retain the name *R. quinquecostatus*, referring to *R. quinquecostatus* sensu Holzinger et al. (2003), to avoid confounding effects of recent taxonomic changes.

This polyphagous planthopper has been recorded in Europe on elms (*Ulmus* spp.), *Salix cinerea*, tall herbaceous vegetation and low-growing shrubs, while in Serbia *R. quinquecostatus* has been found in meadows, grasslands and on woody plants including *Quercus*, *Prunus*, and *Crataegus* species (Janković, 1975; Nickel, 2003; Trivellone et al., 2005; Kosovac et al., 2023b, c). Adults have also been reported in Serbia on several cultivated plants, including maize, grapevine, and potato (Jović et al., 2009; Cvrković et al., 2014; Mitrović et al., 2016). Apart from limited molecular evidence documenting nymphal presence in the rhizosphere of perennial grassland plants (Kosovac et al., 2015), the life history of *R. quinquecostatus* remains largely unknown, leaving unresolved the origin of CaPsol-infected adults colonizing agricultural crops. This knowledge gap is particularly important given the increasing recognition of this planthopper as a CaPsol vector associated with sugar beet RTD and its suspected involvement in maize redness and tobacco stolbur (Kosovac et al., 2023a, b). Furthermore, the species has also been shown to transmit both the 16SrXII-P phytoplasma and the γ-proteobacterium ‘*Candidatus* Arsenophonus phytopathogenicus’ (Kreitzer et al., 2025), pathogens causing major losses in sugar beet and potato production across Europe (Sémétey et al., 2007; Behrmann et al., 2021; Mahillon et al., 2022; Duduk et al., 2024; Rinklef et al., 2024).

Research on sugar beet RTD in Serbia between 2018 and 2023 initially focused on the cixiid planthopper *Pentastiridius leporinus*, the principal vector of ‘*Ca*. A. phytopathogenicus’ associated with SBR in sugar beet in Western European sugar beet-wheat rotations (e.g. Gatineau et al., 2001, 2002; Bressan et al., 2008; Behrmann et al., 2022). However, surveys conducted in RTD-affected plots at the Institute of Field and Vegetable Crops Novi Sad (IFVCNS) experimental fields in Rimski Šančevi failed to detect *P. leporinus* adults or planthopper nymphs associated with sugar beet (Kosovac et al., 2023a), thereby redirecting attention toward other planthopper species. Among these, *R. quinquecostatus* was frequently recorded on weeds, sugar beet (Kosovac et al., 2023a), parsnip (Duduk et al., 2023a), and occasionally cereals (Ćurčić, Ž., unpublished), often co-occurring with *R. panzeri* and, less frequently *R. cuspidatus*, without revealing consistent distribution pattern.

During the same period, CaPsol occurrence in the four major crops in the rotation system at this site: sunflower, wheat, sugar beet, and maize, was unevenly investigated. Sugar beet consistently exhibited RTD symptoms of variable severity, while maize redness occurred sporadically. In contrast, sunflower and wheat were not tested for CaPsol due to the absence of characteristic phytoplasma symptoms. Despite its widespread cultivation in Serbia, covering more than 240, 000 ha (Statistical Office of the Republic of Serbia, 2025), and its prominent role in cereal-based rotations (Crnobarac et al., 2016), sunflower has received little attention as a potential CaPsol reservoir in local epidemiological studies. Although sunflower was identified as a CaPsol host in Bulgaria (Avramov et al., 2016), its epidemiological relevance in Serbia has likely been overlooked due to the absence of clearly described disease symptoms or limited attention to symptom recognition, particularly floral deformities and seed sterility.

While crop rotations are known to influence pathogen and vector dynamics, their role in structuring *Reptalus* spp. communities and sustaining CaPsol cycles through in-field vector development remains unclear. Together, the previously described crop-associated CaPsol epidemiological cycle involving *R. panzeri*, the unresolved life history of *R. quinquecostatus*, and the limited assessment of asymptomatic crops suggest that cultivated hosts may play a broader role in CaPsol epidemiology than currently recognized. This study therefore aimed to elucidate the origin and crop associations of *Reptalus* spp. within a four-crop rotation system at the IFVCNS experimental fields, with particular focus on *R. quinquecostatus* and its linkage to rotational crop sequences in Serbia. We hypothesized that (i) planthopper species exhibit crop-dependent developmental associations within the rotation system and (ii) asymptomatic crops may function as cryptic CaPsol reservoirs sustaining pathogen persistence. To address these questions, we integrated field surveys of planthopper nymphs, molecular species identification, adult monitoring, and emergence-cage experiments. In parallel, an epidemiological assessment of the sunflower-wheat sequence was conducted to evaluate CaPsol persistence within the rotation and identify the vector species responsible for its maintenance under field conditions.

## 2 Materials and methods

### 2.1 Study site, crop rotation system and experimental design

**Figure 1:**
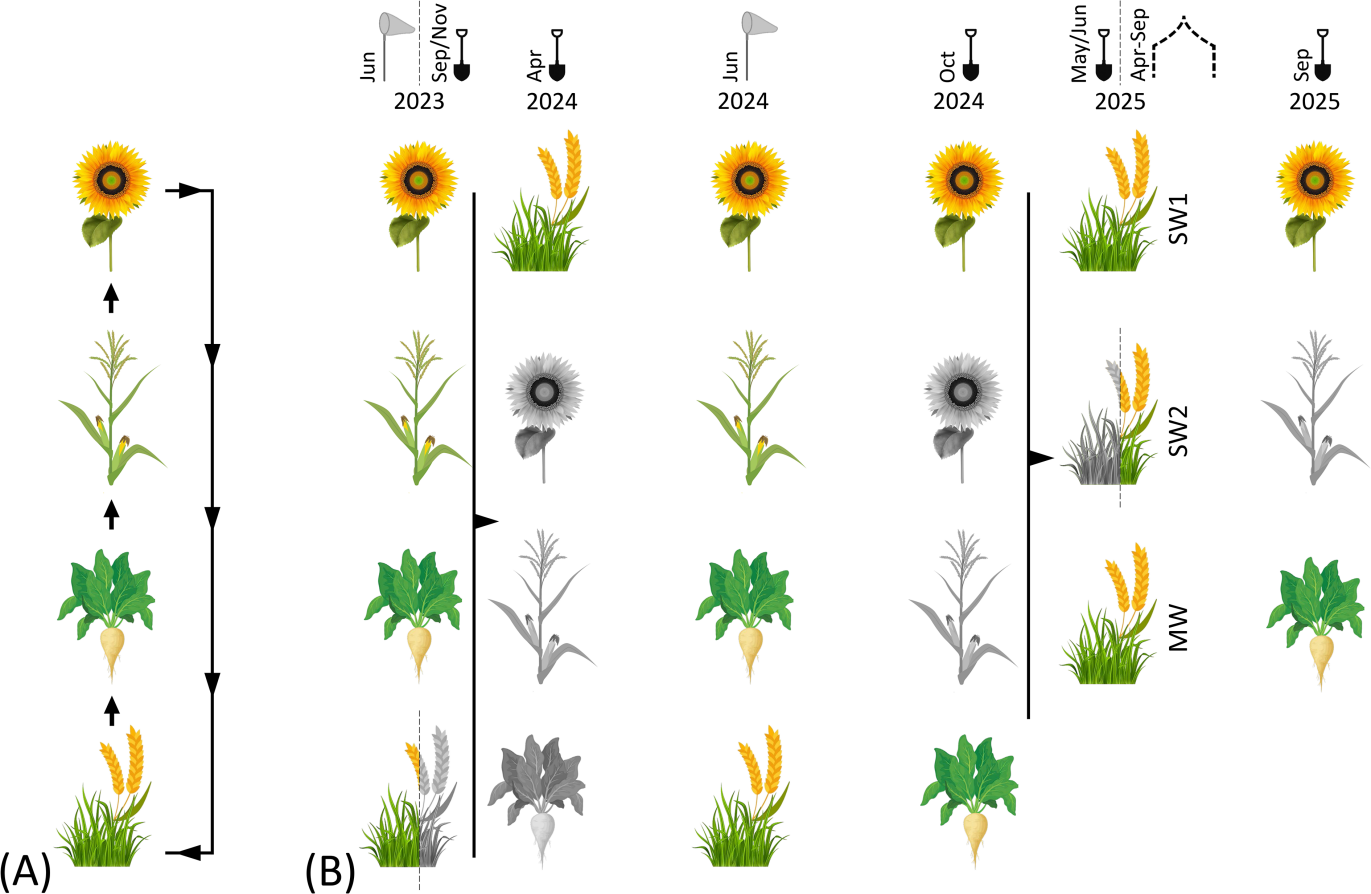
Crop rotation system and sampling scheme applied in the study. **(A)** Long-established four-crop rotation at the IFVCNS experimental site; **(B)** Overview of crop sequences and planthopper assessments conducted during 2023-2025, with sampling phases and methods indicated by specific symbols. Crops shown fully or partially in grey denote phases when planthopper sampling was not performed.

A three-year study (2023-2025) was conducted at the IFVCNS experimental fields in Rimski Šančevi (45°19′59.3″N, 19°49′53.3″E), in the Bačka Region of northern Serbia, at approximately 84 m a.s.l. The site is characterized by chernozem soil and a temperate continental climate (mean annual temperature ~11 °C; precipitation ~620 mm). This agroecosystem consists of a long-established four-crop rotation system (sunflower-wheat-sugar beet-maize) maintained under open-field conditions for more than six decades (**Figure 1A**). Following the terminology of Ballot et al. (2023), the term *crop sequence* refers in our study to the order of crops grown consecutively within monitored plots during the study period, whereas *crop rotation* refers to the broader recurring multi-year agronomic system.

The study began in summer 2023 and covered two complete cropping seasons (2024-2025), concluding in autumn 2025 at crop maturity (**Figure 1B**). In 2023, adult planthoppers were surveyed in June across all four crops, while potential oviposition hosts (sunflower, maize, and sugar beet) were assessed by thorough root inspections in September and November. In 2024, nymphs were surveyed in April in wheat following sunflower, adults were monitored in June in all four crops, and root inspections were conducted in sunflower and sugar beet plots in October. An additional maize-wheat sequence was established during 2024/2025 to evaluate its relevance for life cycle of *R. quinquecostatus*. In May and June 2025, nymphs were surveyed in wheat following both sunflower and maize, adult emergence was monitored from April to September using emergence cages, and final root inspections were conducted in September in sunflower and sugar beet plots adjacent to wheat.

### 2.2 Planthopper sampling and emergence monitoring

Planthopper nymphs and adults were sampled across eight phases using life stage-appropriate methods, including soil digging, sweep-net transects, and emergence-cages (**Figure 1B**). Nymphs were surveyed on sunflower, maize, and sugar beet roots during autumn (2023-2025), and on wheat roots during spring (2024-2025) in plots previously cultivated with sunflower or maize. In each plot, ten soil blocks (30 × 30 × 30 cm) were sampled along a diagonal transect. Samples were collected at the base of individual plants in rows or from row sections (~30 plants in case of wheat). Nymphs were collected using aspirators and preserved in 96% ethanol.

Adult planthoppers were sampled in June 2023 and 2024 across all four crops. In sunflower and maize, adults were collected directly from plants, whereas sweep-net sampling was used in sugar beet and wheat. In 2023, one plot per crop was surveyed along a single diagonal transect. Sampling intensity was increased in 2024 using crop-adapted transect designs (radial in sunflower and maize, line in sugar beet, and belt in wheat; Popov et al., 2024) to ensure representative coverage. Surveys were conducted in eight rounds at twice-weekly intervals.

Emergence of adults was monitored from April to September 2025 using mesh field cages (1.5 × 4 × 2 m) installed over three wheat plots: two following sunflower (SW1 and SW2) and one following maize (MW) (**Figure 1B**). Each cage contained a yellow sticky trap to detect first adult emergence. Traps were inspected twice weekly until mid-May and daily thereafter. Following the first adult capture, monitoring continued through direct cage inspections conducted three times weekly until late July, twice weekly in August, and weekly until late September, when cages were dismantled. Adults were collected by sweeping wheat plants within cages and aspirating individuals from the cage mesh.

All collected adults, including specimens removed from sticky traps using limonene, were preserved in 96% ethanol and stored at 4 °C until further analysis.

### 2.3 Morphological and molecular identification of planthoppers

Nymphs were identified using molecular markers, whereas adults were assigned to species based on a combination of morphological and molecular criteria (Holzinger et al., 2003; Bertin et al., 2010a, 2010b; Pfitzer et al., 2022). Nymphal instars were determined according to descriptions of *H. obsoletus* immatures based on antennal and metatarsal development (instars N1-N2), as well as metatarsomere number and wing pad development (N3-N5) (Sforza et al., 1999; Cargnus et al., 2012). Genomic DNA was extracted from individual specimens using a modified CTAB protocol (Gatineau et al., 2001). PCR assays were performed in 25 µL reaction volumes containing 1 µL of template DNA, 1×Taq PCR Master Mix (EURx Sp., Poland), and 0.5 µM of each primer under previously published thermal conditions. Amplicons were subjected to RFLP analysis when required and visualized on 2% agarose gels under UV illumination.

Molecular identification followed a stepwise approach. Nymphs were initially screened using *P. leporinus*-specific primers (Pfitzer et al., 2022), with DNA from a male *P. leporinus* specimen collected on sugar beet in Baden-Württemberg, Germany, used as a positive control (kindly provided by A. Rinklef, Fraunhofer IME, Giessen, Germany). Samples that failed to amplify were subsequently analyzed using ITS2 and mitochondrial COI markers to detect *Reptalus* and *Hyalesthes* planthoppers and distinguish species within these genera (Bertin et al., 2010a, b). DNA from target planthopper species deposited in the A. Kosovac and M. Šćiban entomological collections served as positive controls.

Adults were assigned to genus based on external morphology. Species-level identification was performed using male genitalia examined under a stereomicroscope (STM-8; BTC, Hungary), whereas females of *R. quinquecostatus* and *R. panzeri* were differentiated using molecular assays (Holzinger et al., 2003; Bertin et al., 2010a).

### 2.4 Detection and genotyping of CaPsol in planthoppers and plant hosts

All nymphs collected between 2023 and 2025, as well as adult planthoppers from populations used in transmission experiments, including 24 *R. quinquecostatus* individuals per population (2024-2025), were screened for CaPsol. Within the sunflower-wheat sequence, both crops were assessed for CaPsol presence in 2024 and 2025. In April 2024, 30 wheat root samples were collected from a plot previously cultivated with sunflower, while 24 sunflower leaf samples were collected in August from an adjacent plot, following the sampling approaches described by Jović et al. (2009) and Avramov et al. (2016), respectively. In 2025, 60 wheat root samples (30 per plot) were collected in early June from two emergence-cage plots (SW1 and SW2), and 30 sunflower root samples were collected in late August from a plot adjacent to SW1. All plant samples were collected from asymptomatic plants along diagonal transects.

Genomic DNA from plant tissues was extracted using the CTAB protocol (Doyle and Doyle, 1990). DNA concentration and purity were determined using a NanoDrop™ 2000 spectrophotometer (Thermo Scientific™, USA). Plant DNA was diluted 1:30 prior to qPCR and nested PCR, while sunflower samples were additionally tested at a 1:100 dilution to reduce potential PCR inhibition.

Phytoplasma detection was performed using two TaqMan qPCR assays targeting the 16S rRNA gene: a universal phytoplasma assay and a 16SrXII-specific assay detecting both CaPsol (16SrXII-A) and 16SrXII-P phytoplasmas without lineage discrimination (Christensen et al., 2004, 2013; Behrmann et al., 2022). The eukaryotic 28S rRNA gene (UNI28S) was used as an internal control (Mittelberger et al., 2020). Reactions were carried out in 12 µL volumes using the SensiFAST™ Probe No-ROX Kit (Bioline, USA) on a CFX Opus 384 Real-Time PCR System (Bio-Rad, USA). Primers and probes were used at a final concentration of 0.4 µM and 0.2 µM, respectively. Cycling conditions were adapted according to the manufacturer’s recommendations with an initial 2 min at 50 °C and 5 min at 95 °C, followed by 40 cycles of 10 s at 95 °C and 50 s at 60 °C. Data were analysed using Bio-Rad CFX Maestro software, version 2.3 (Bio-Rad, USA). Samples were considered positive when exponential amplification was detected at Cq values <37 in the universal assay and <35 in the 16SrXII-specific assay (Behrmann et al., 2022). As the universal phytoplasma qPCR assay of Christensen et al. (2004, 2013) does not define a fixed positivity threshold, the cutoff value was established based on internal assay validation, reproducibility among replicates, and consistent exponential amplification near the assay detection limit. Each run included no-template controls, phytoplasma-free insect or sugar beet DNA as negative controls, and DNA from reference phytoplasma strains from laboratory collection as positive controls.

Assignment to CaPsol (16SrXII-A) was confirmed by nested PCR targeting the *stamp* gene (Fabre et al., 2011), which does not amplify 16SrXII-P strains (Duduk et al., 2023b). Positive controls included 16SrXII-A DNA from sugar beet and 16SrXII-P DNA from periwinkle obtained from a reference phytoplasma collection (kindly provided by Dr B. Jarausch, JKI, Siebeldingen, Germany). Samples were considered CaPsol-positive when amplification was obtained with both qPCR assays and the *stamp* assay. Samples yielding inconsistent results were further analysed by nested PCR targeting the 16S rRNA gene (P1/P7 followed by R16F2n/R16R2) to verify phytoplasma presence (Deng and Hiruki, 1991; Lee et al., 1995; Schneider et al., 1995).

CaPsol genotyping based on the *stamp* gene was performed for all infected nymphs collected in 2024 and 2025, randomly selected *R. quinquecostatus* adults (10 specimens per year), and CaPsol-positive sunflower samples (10). *Stamp* amplicons were bidirectionally sequenced (Macrogen Inc., South Korea), edited in FinchTV v1.4.0 (Geospiza Inc., Seattle, USA), and aligned with GenBank reference sequences using ClustalW implemented in MEGA X (Kumar et al., 2018). The same subset of samples was further analysed by *tuf* profiling using nested PCR with primer pairs fTuf1/rTuf1 and fTufAY/rTufAY, followed by RFLP analysis with *Hpa*II and *Tai*I and fragment separation on 8% polyacrylamide gels (Langer and Maixner, 2004; Ćurčić et al., 2021a). Representative *stamp* gene sequences from each host were deposited in the GenBank database under accession numbers PZ289166-PZ289193.

### 2.5 CaPsol transmission experiments with *R. quinquecostatus*

To evaluate the role of *R. quinquecostatus* in CaPsol epidemiology within the sunflower-wheat sequence, transmission experiments were conducted in 2024 and 2025 using sunflower (cv. NS Kruna) and wheat (cv. Pobeda). Sugar beet (cv. Original) was included to evaluate transmission across crops differing in epidemiological relevance, while periwinkle (*Catharanthus roseus*) served as a control host. Planthoppers used in the trials were collected in late May 2024 from wheat following sunflower and in early June 2025 from the SW1 plot. In 2024, sunflower (6 replicates), sugar beet (11), and periwinkle (5) plants were inoculated with naturally CaPsol-infected *R. quinquecostatus*, while in 2025, sunflower (7 replicates) and wheat (9) were included in the experiment. Plants were grown in pathogen-free substrate under controlled conditions (24 ± 1 °C; 16:8 h light/dark). Sunflower, sugar beet, and periwinkle plants were treated as individual replicates, while each wheat replicate consisted of three pooled plants.

Prior to inoculation tests each year, 24 insects were identified to species level and screened for CaPsol. Plants were enclosed in ventilated cylinders and exposed to ~30 insects per replicate for 72 h, after which insects were removed and preserved in 96% ethanol. One month after inoculation, sunflower, wheat, and sugar beet plants were transplanted into larger pots and maintained under controlled indoor conditions. Negative controls (three plants per species) were included each year. Sampling was conducted three months post-inoculation or earlier in cases of plant decline. Root tissues from sunflower, wheat, and sugar beet, together with leaf tissues from periwinkle, were collected for CaPsol detection and genotyping as described previously.

Planthopper identity was verified after completion of the transmission experiments. Replicates containing species other than *R. quinquecostatus* were treated as non-target inoculations. Transmission results were therefore evaluated primarily based on replicates containing exclusively *R. quinquecostatus* individuals to eliminate potential interference from other species.

### 2.6 Semi-field CaPsol transmission experiment to sunflower

In 2025, the role of *R. quinquecostatus* as a vector of CaPsol to sunflower was additionally evaluated under semi-field conditions. On a sunflower plot adjacent to SW1, 25 plants (cv. NS Kruna) were enclosed within a mesh cage (1.5 × 4 × 2 m), while a second cage with 24 plants under identical conditions served as a negative control and was equipped with a yellow sticky trap to monitor planthopper presence. This single-replicate setup was employed as an exploratory transmission assay to complement individual plant transmission trials. Sunflower was sown in mid-April and maintained under standard agronomic practices until cage installation in mid-May. In mid-June, 200 field-collected adult planthoppers from SW1 were released into the experimental cage with sunflower plants at the 8–10 leaf stage. Cages were inspected weekly for symptom development and remained in the field until late September. At the end of the experiment, root tissues from inoculated and control plants were collected for CaPsol detection and molecular characterization.

### 2.7 Small-scale study of *H. obsoletus* role in sunflower-associated CaPsol epidemiology

A sunflower plot adjacent to SW2, characterized by high *H. obsoletus* abundance and sporadic occurrence of *Reptalus* spp. during June-July 2025, was selected as a small-scale study site to investigate CaPsol dynamics associated with sunflower and *H. obsoletus* as an alternative vector. A total of 24 *H. obsoletus* adults collected from *C. arvensis* within the assessed sunflower plot were screened for CaPsol, and 10 of those individuals were randomly selected for pathogen genotyping. Transmission experiments were conducted using field-collected *H. obsoletus* adults, with experimental sunflower (8 replicates), sugar beet (6), and periwinkle (2) plants each exposed to ~30 insects per replicate during the inoculation trials. Sugar beet was included to enable comparison of transmission efficiency, whereas periwinkle served as a control host. In late August, sunflower root samples (30) and stem samples from asymptomatic *C. arvensis* plants (20) were collected along diagonal transect within the plot. CaPsol detection was performed as described previously, with ten positive samples from each host plant selected for genotyping.

## 3 Results

### 3.1 Species and instar composition of planthopper nymphs

**Figure 2:**
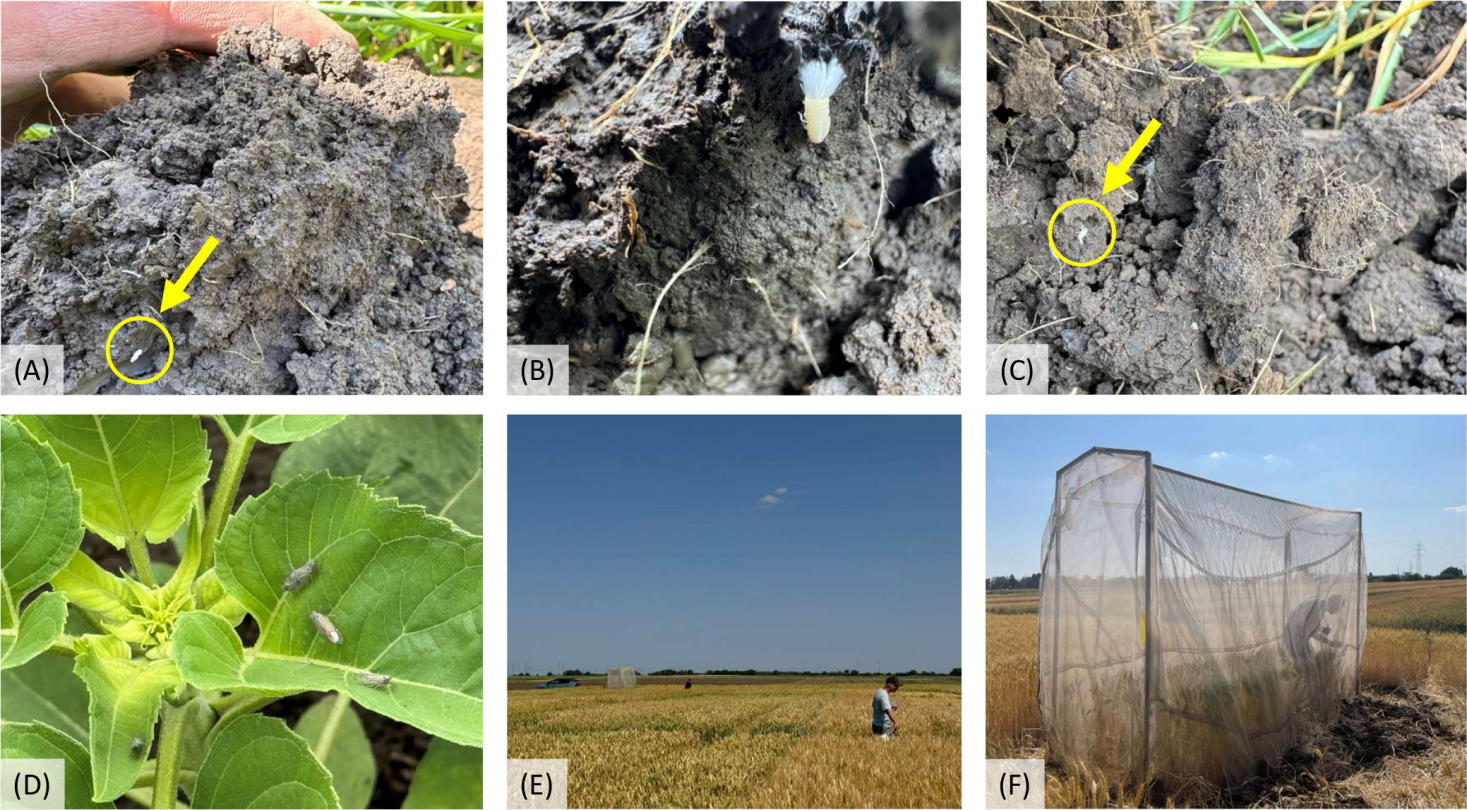
Occurrence and field sampling of planthoppers at IFVCNS experimental site (2024-2025). Nymphs on crop roots: **(A)** wheat after sunflower (April 2024), **(B)** sunflower (October 2024), and **(C)** wheat after maize (June 2025). Adult monitoring and sampling in mid-June 2025: **(D)** *Reptalus* spp. adults on sunflower foliage; **(E)** adults collected from wheat in SW1 for transmission assays, and **(F)** inspection of emergence cage in the SW1 plot.

**Figure 3:**
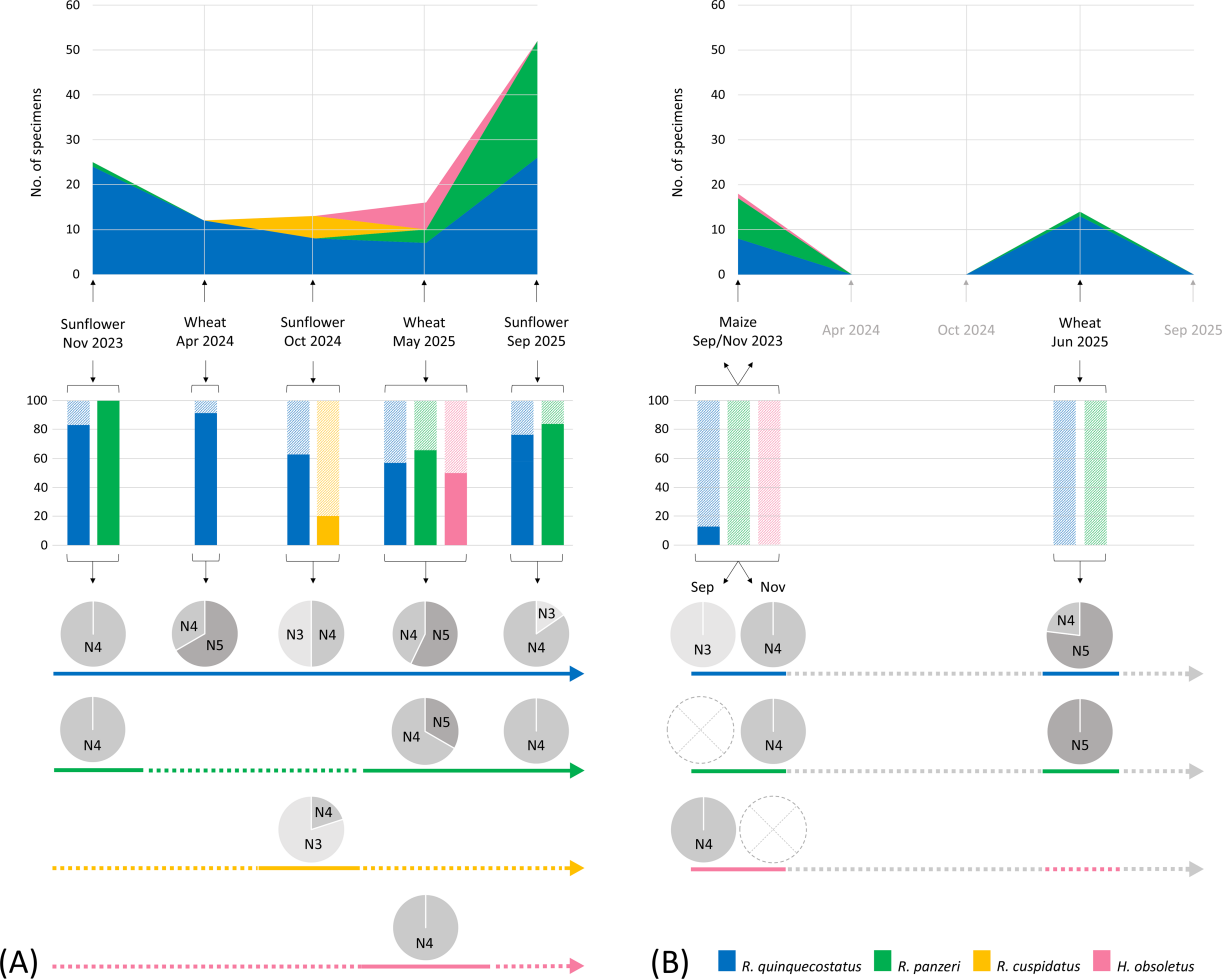
Seasonal dynamics of planthopper nymphs: species diversity, CaPsol infection status, and nymphal instar composition in the **(A)** sunflower-wheat and **(B)** maize-wheat sequences. Upper panels show nymph abundance and species diversity; middle panels show proportions of CaPsol-infected (solid) and non-infected (patterned) individuals (%); lower panels present relative instar composition (N3-N5, %). Grey month labels on upper panels indicate unassessed sampling phases. Colors correspond to species as defined in the legend.

The sunflower-wheat sequence consistently supported planthopper nymph populations throughout the three-year study period (2023-2025), whereas the maize-wheat sequence, evaluated non-consecutively in 2023 and 2025, showed comparable, but generally lower, nymphal abundance (**Figures 2A–C**, **3**). No nymphs were detected on sugar beet roots during the study. Molecular screening excluded the presence of *P. leporinus*, and all collected nymphs were identified as *Reptalus* or *Hyalesthes* species.

Within the sunflower-wheat sequence, *R. quinquecostatus* predominated on sunflower roots across all sampling phases, accounting for 24/25 individuals in November 2023, 8/13 in October 2024, and 26/52 in September 2025 (**Figure 3A**). A similar pattern was observed in wheat following sunflower, where all nymphs collected in April 2024 (12/12) and nearly half collected in May 2025 (7/16) belonged to this species. Among the remaining individuals, *R. panzeri* occurred at low frequency in most samplings (1/25 in November 2023; 3/16 in May 2025), but reached equal proportions to *R. quinquecostatus* on sunflower in September 2025 (26/52). Nymphs of *R. cuspidatus* were detected only on sunflower in October 2024 (5/13), whereas *H. obsoletus* was recorded only in wheat following sunflower in May 2025 (6/16).

Fewer nymphs were collected within the maize-wheat sequence than in the sunflower-wheat sequence at comparable sampling periods (**Figure 3B**). On maize roots in September and November 2023, *R. quinquecostatus* and *R. panzeri* occurred in similar proportions (8/18 and 9/18, respectively), together with a single *H. obsoletus* individual. In wheat following maize (June 2025), *R. quinquecostatus* clearly predominated (13/14), while only one *R. panzeri* nymph was detected, and *R. cuspidatus* was absent.

Three nymphal instars (N3-N5) of *R. quinquecostatus* were identified in both crop sequences. On sunflower, N3 instars were recorded in small numbers in October 2024 (4/8) and September 2025 (4/26), whereas N4 predominated across all autumn samplings, including all individuals collected in November 2023 (24/24). In wheat following sunflower, N5 was the dominant instar in April 2024 (8/12) and May 2025 (4/7), while the remaining individuals belonged to N4 (**Figure 3A**). A similar developmental pattern was observed in the maize-wheat sequence, where N3 instars occurred on maize in September 2023 (5/5), progressed to N4 in November (3/3), and predominantly reached N5 in wheat by June 2025 (10/13) (**Figure 3B**).

For *R. panzeri*, N4 nymphs predominated on sunflower roots in autumn. The single individual recorded in November 2023 and all individuals collected in September 2025 (26/52) belonged to this instar. In wheat following sunflower, both N4 and N5 instars were recorded in May 2025 (2/3 and 1/3, respectively), whereas the single nymph recorded in wheat following maize in June 2025 belonged to N5 (**Figure 3**).

Nymphs of *R. cuspidatus* collected from sunflower in October 2024 were predominantly N3 instars (4/5), with one N4 individual recorded. All *H. obsoletus* nymphs were identified as N4, including those collected from wheat following sunflower in May 2025 (6/6) and the single specimen recorded on maize in September 2023 (**Figure 3**).

### 3.2 CaPsol infection and strain diversity in nymphs

Nymphs of all recorded planthopper species tested positive for CaPsol, although infection rates differed markedly between crop sequences (**Figure 3**; **Table 1**). Within the sunflower-wheat sequence, infection levels were consistently high. Nymphs of *R. quinquecostatus* collected from sunflower roots showed infection rates ranging from 62% to 83%, with co-occurring *R. panzeri* collected in 2025 showing similarly high infection levels (85%). Nymphs collected from wheat following sunflower were also highly infected, with *R. quinquecostatus* reaching 92% positivity (11/12) in April 2024. CaPsol was additionally detected in *R. cuspidatus* collected from sunflower (20%; 1/5) and in *H. obsoletus* collected from wheat following sunflower (50%; 3/6) (**Figure 3A**).

**Table 1:** Diversity of CaPsol strains in planthopper nymphs from the sunflower-wheat sequence (2023-2025) based on *stamp* and *tuf* genotyping.

Crop	Sampling phase	Planthopper species	CaPsol-positive/analysed	CaPsol genotype (n)
Sunflower	November 2023	*R. quinquecostatus*	20/24	NA
		*R. panzeri*	1/1	
	October 2024	*R. quinquecostatus*	5/8	STOL(St4)/tuf-d (5)
		*R. cuspidatus*	1/5	STOL(St4)/tuf-d (1)
	September 2025	*R. quinquecostatus*	20/26	STOL(St4)/tuf-d (20)
		*R. panzeri*	22/26	STOL(St4)/tuf-d (22)
Wheat	April 2024	*R. quinquecostatus*	11/12	STOL(St4)/tuf-d (11)
	May 2025	*R. quinquecostatus*	4/7	STOL(St4)/tuf-d (3)
				BG4560(St31)/tuf-b (1)
		*R. panzeri*	2/3	STOL(St4)/tuf-d (2)
		*H. obsoletus*	3/6	STOL(St4)/tuf-d (2)
				Rqg50(St1)/tuf-b (1)

NA, not analysed. *Stamp* genotype nomenclature follows Kosovac et al. (2023a) with integrated previously published data. Reference accession numbers for *stamp* genotypes: Rqg50 (St1; KC703019), STOL (St4; FN813261), BG4560 (St31; FN813252).

In contrast, infection levels within the maize-wheat sequence were low (**Figure 3B**). In 2023, only a single *R. quinquecostatus* nymph collected from maize roots tested positive for CaPsol (1/8), while all *R. panzeri* and *H. obsoletus* individuals were negative. No CaPsol was detected in nymphs collected from wheat in June 2025.

CaPsol strain characterization revealed a clear predominance of the STOL(St4)/tuf-d genotype in nymphs associated with the sunflower-wheat sequence, regardless of host crop, sampling phase, or vector species (**Table 1**). Limited CaPsol strain heterogeneity was observed only in nymphs collected from wheat in April 2025, where genotype BG4560(St31)/tuf-b was detected in *R. quinquecostatus* (1/4) and genotype Rqg50(St1)/tuf-b in *H. obsoletus* (1/3).

### 3.3 Species composition of planthopper adults

**Figure 4:**
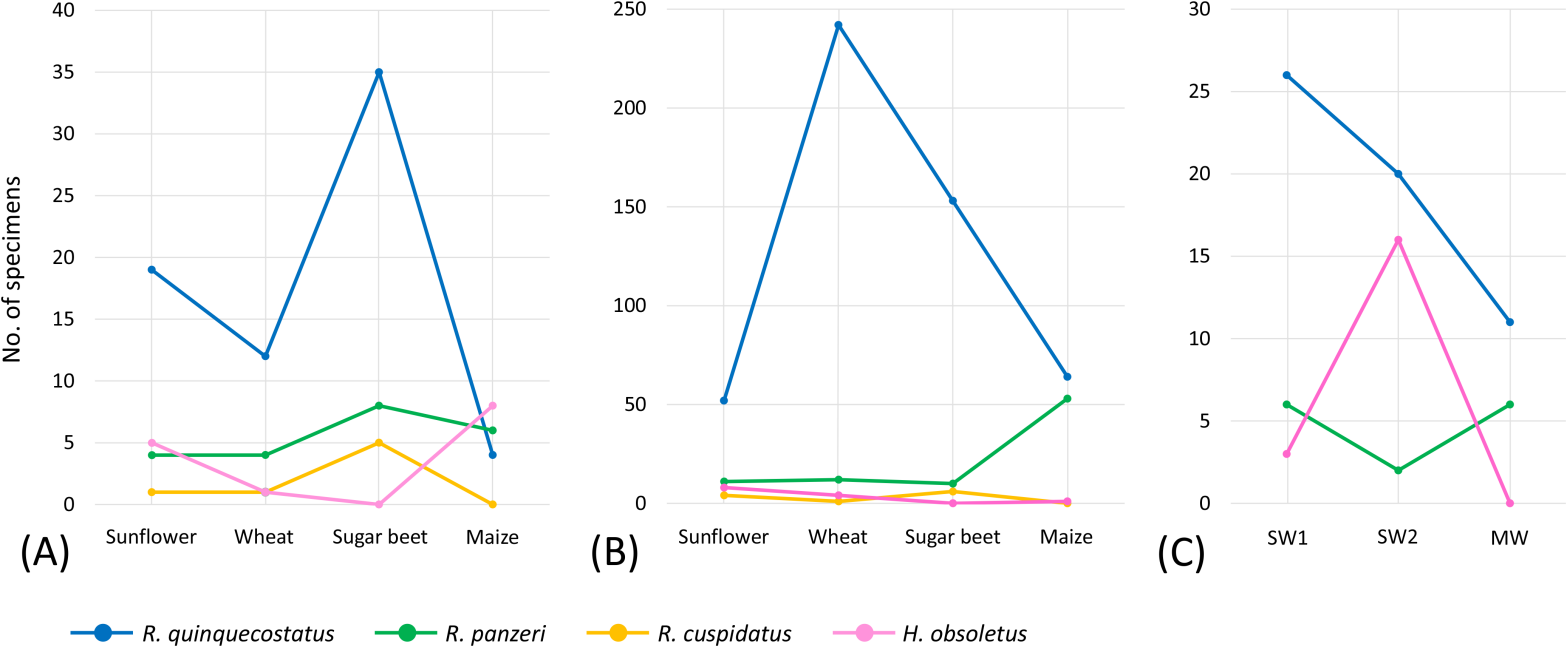
Adult planthopper species composition and abundance across crops and emergence cages. **(A)** Adults of both sexes collected in June 2023; **(B)** Male individuals collected in June 2024; **(C)** Adults of both sexes emerging in 2025 in cages installed in wheat following sunflower (SW1, SW2) and maize (MW). The y-axis indicates the number of individuals (note differing scales across panels **(A–C)** to improve visualization). Colors correspond to species as defined in the legend.

During adult monitoring conducted in June 2023 and 2024, four planthopper species were recorded across the assessed crops: *R. quinquecostatus*, *R. panzeri*, *R. cuspidatus*, and *H. obsoletus* (**Figures 2D, E**; **Figure 4**). In 2023, *R. quinquecostatus* predominated across crops accounting for 61.9% of all collected individuals (70/113) (**Figure 4A**). The species was most abundant in sugar beet (35 individuals), followed by sunflower (19) and wheat (12), while only a few individuals were recorded in maize (4). A proportion of 19.5% of the total catch (22 individuals) was attributed to *R. panzeri*, which also occurred in all crops, with highest abundance in sugar beet (8) and maize (6), and equal numbers in sunflower and wheat (4 each). Only 6.2% of specimens (7 individuals) were identified as *R. cuspidatus*, occurring mainly in sugar beet (5), with single individuals recorded in sunflower and wheat, and none in maize. The remaining 12.4% of collected specimens (14 individuals) belonged to *H. obsoletus*, which was most abundant in maize (8) and sunflower (5), with only one individual recorded in wheat and none in sugar beet.

Because substantially higher numbers of adults were collected in 2024 as a result of the more intensive sampling scheme, species identification was restricted to males. A total of 621 males were identified across the four crops (corrected data from Popov et al., 2024) (**Figure 4B**). The community was dominated by *R. quinquecostatus*, which accounted for 82.3% of all identified individuals. The species was most abundant in wheat following sunflower (242 individuals), followed by sugar beet (153), maize (64), and sunflower (52). In contrast, *R. panzeri* occurred at lower frequencies (13.9%) and was most abundant in maize (53), with fewer individuals recorded in wheat (12), sunflower (11), and sugar beet (10). The remaining species were rare: *R. cuspidatus* represented 1.7% of the identified specimens and occurred sporadically in sugar beet (6), sunflower (4), and wheat (1), but was absent from maize, while *H. obsoletus* accounted for 2.1% of the total catch and was recorded mainly in sunflower (8) and wheat (4), with a single individual in maize and none in sugar beet.

Adult planthopper emergence monitoring conducted in cages in 2025 revealed differences among crop-sequence plots (**Figures 2F**, **4C**). The highest numbers of emerged planthoppers were identified as *R. quinquecostatus*, with 26, 20, and 11 individuals recorded in SW1, SW2, and MW, respectively. Lower numbers of *R. panzeri* were recorded in SW1 and MW (6 individuals each), while only two individuals were recorded in SW2. Emergence of *H. obsoletus* was recorded in wheat following sunflower, with three individuals collected in SW1 and 16 in SW2, whereas no individuals were detected in the MW cage.

### 3.4 CaPsol epidemiology within the sunflower-wheat sequence

**Figure 5:**
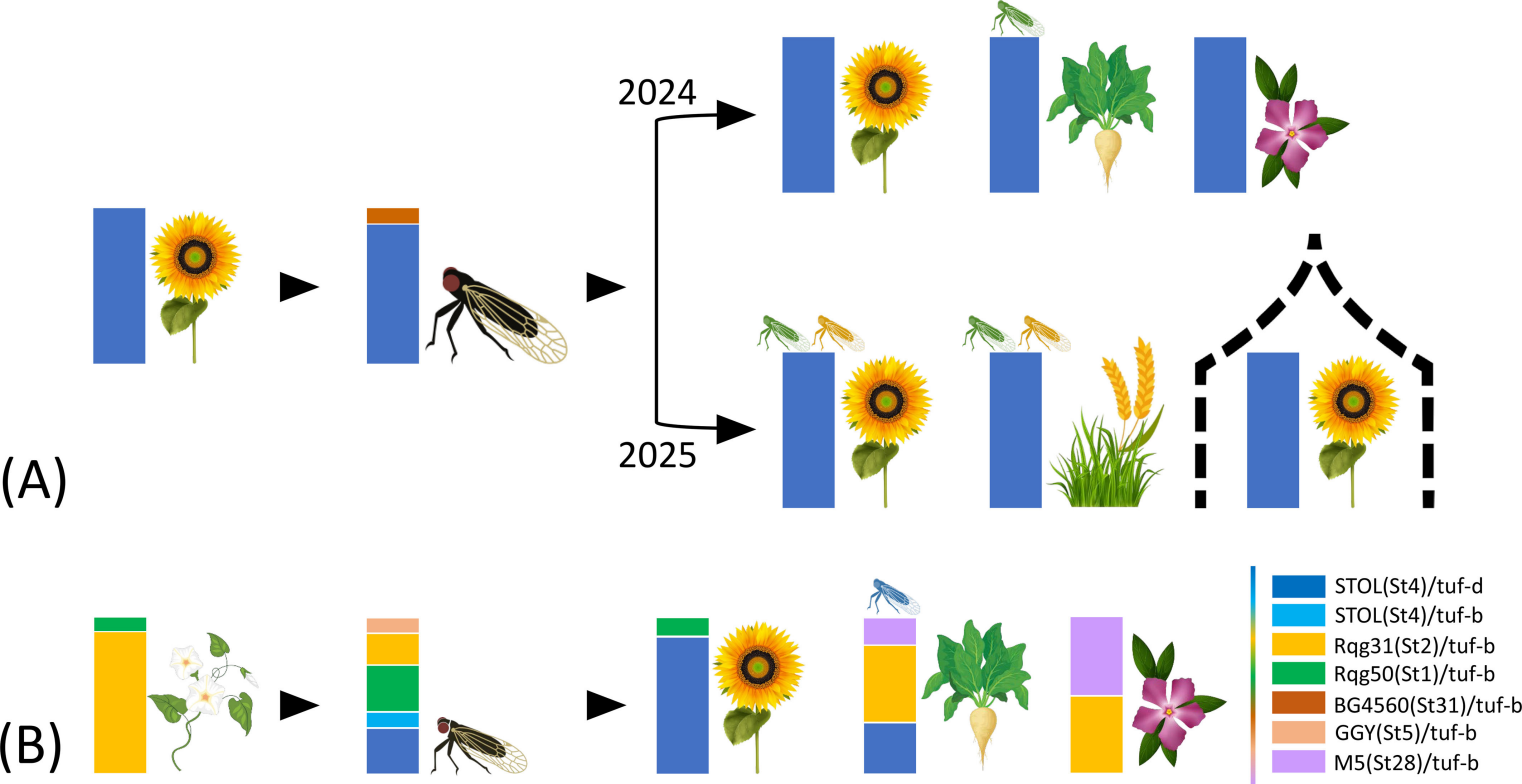
CaPsol strain diversity in host plants, vector populations, and experimental plants. **(A)** Strain composition in the sunflower-*R. quinquecostatus* pathosystem (2024–2025), integrating CaPsol diversity in *R. quinquecostatus* and experimental plants across both years. **(B)** Strain composition in the *C. arvensis*-*H. obsoletus* pathosystem (2025). Plants and vectors are represented schematically. The presence of non-target planthoppers, *R. quinquecostatus, R. panzeri*, and *R. cuspidatus*, is indicated by blue, green, and yellow insect symbols, respectively, adjacent to the affected plants. Bars indicate CaPsol strain frequencies (%) based on *stamp* and *tuf* typing, with colors corresponding to genotypes defined in the legend.

Leaf samples collected from sunflower in 2024 tested negative for CaPsol (0/24), whereas root samples collected in 2025 showed 100% CaPsol incidence based on both qPCR assays (30/30). Nested PCR showed lower sensitivity, with amplification detected in only 6/30 samples at a 1:30 DNA dilution. Increasing the template DNA dilution to 1:100 improved amplification efficiency, yielding 11 additional positive samples (*stamp* and *tuf*) thereby increasing concordance with qPCR to 57% (17/30 samples). All samples negative for *stamp* were also negative for *tuf* and 16S rRNA nested PCR. Genotyping of all analysed strains identified the STOL(St4)/tuf-d genotype (**Figure 5A**).

CaPsol was not detected in wheat samples collected in 2024 (0/30 samples), nor in samples collected from the SW1 and SW2 plots in 2025 (0/60).

Analysis of the 2024 *R. quinquecostatus* population indicated a high CaPsol infection rate (96%; 23/24), with the majority of genotyped strains (9/10) assigned to STOL(St4)/tuf-d and one to BG4560(St31)/tuf-b (**Figure 5A**). In 2025, all analysed individuals were identified as *R. quinquecostatus*, with a lower infection rate (58%; 14/24), and all characterized strains belonged to STOL(St4)/tuf-d genotype.

Experimental plants were exposed to 27–32 insects per replicate (**Table 2**). In 2024, the first *R. quinquecostatus* adults were recorded during week 21 (May) in wheat following sunflower, and adults from this population were subsequently used in transmission experiments. CaPsol transmission was highly efficient, reaching 100% in sunflower (6/6 plants) and 90% in sugar beet (10/11). All infected plants harbored the STOL(St4)/tuf-d genotype. All periwinkle plants were also infected (5/5) and carried the same genotype (**Figure 5A**; **Table 2**). One infected sugar beet replicate resulted from unintended inoculation involving two *R. panzeri* females present alongside *R. quinquecostatus*, one of which tested positive for CaPsol (replicate Sb20-24, **Table 2**).

**Table 2:** CaPsol-positive experimental plants obtained in transmission trials (2024–2025) with *Reptalus spp*.

Year	Experimental plant; CaPsol-positive/total replicates	Replicate ID	Planthoppers in transmission trial CaPsol-positive/total individuals^*^						CaPsol genotype *stamp*/*tuf*
			♂ Rq	♀ Rq	♂ Rp	♀ Rp	♂ Rc	♀ Rc	
2024	Sunflower 6/6	Sf2-24	18	13	/	/	/	/	STOL(St4)/tuf-d
		Sf3-24	18	9	/	/	/	/	
		Sf4-24	16	16	/	/	/	/	
		Sf5-24	21	11	/	/	/	/	
		Sf6-24	20	10	/	/	/	/	
		Sf7-24	18	11	/	/	/	/	
	Sugar beet 10/11	Sb5/24	19	10	/	/	/	/	STOL(St4)/tuf-d
		Sb7-24	18	9	/	/	/	/	
		Sb8-24	19	8	/	/	/	/	
		Sb10-24	23	9	/	/	/	/	
		Sb11-24	24	6	/	/	/	/	
		Sb12-24	17	13	/	/	/	/	
		Sb14-24	25	4	/	/	/	/	
		Sb15-24	23	7	/	/	/	/	
		Sb17-24	18	12	/	/	/	/	
		**Sb20-24**	14	15	/	1/2	/	/	
	Periwinkle 5/5	W1-24	17	12	/	/	/	/	STOL(St4)/tuf-d
		W2-24	17	14	/	/	/	/	
		W3-24	11	18	/	/	/	/	
		W4-24	8	20	/	/	/	/	
		W5-24	11	17	/	/	/	/	
2025	Sunflower 7/7	Sf1-25	21	6	/	/	/	/	STOL(St4)/tuf-d
		Sf3-25	23	7	/	0/1	/	/	
		**Sf4-25**	19	7	0/1	1/3	/	/	
		**Sf5-25**	13	14	0/2	1/2	/	/	
		**Sf6-25**	19	6	1/2	2/4	/	0/1	
		Sf15-25	20	9	/	0/1	/	/	
		Sf16-25	17	13	0/1	0/1	/	/	
	Wheat 2/9	Wh6-25	21	8	0/1	0/1	0/1	/	STOL(St4)/tuf-d
		Wh7-25	11	16	/	/	/	/	

The abbreviations used for planthopper species are: Rq (*R. quinquecostatus*), Rp (*R. panzeri*), and Rc (*R. cuspidatus*); sex symbols indicate the number of male and female specimens present in each replicate. Replicates shown in bold indicate the presence of CaPsol-positive non-target planthoppers during inoculation. (^*^) CaPsol infection is shown only for non-target planthopper individuals per replicate. Corresponding reference accession number for the identified genotype: STOL (St4; FN813261).

In 2025, *R. quinquecostatus* adults were first recorded during week 24 (June) in the SW1 wheat plot. Transmission experiments using insects originating from this population resulted in 100% infection of sunflower plants (7/7). Although non-target planthoppers were present in most replicates (6/7), transmission could be potentially attributed to *R. quinquecostatus* in three replicates (Sf3-25, Sf15-25 and Sf16-25) in which non-target individuals tested negative for CaPsol (**Table 2**). In the remaining three replicates (Sf4-25, Sf5-25 and Sf6-25), CaPsol-positive *R. panzeri* individuals were detected. All infected sunflower replicates carried the STOL(St4)/tuf-d genotype (**Figure 5A**). Transmission to wheat was substantially lower with only 2/9 plants testing positive and both infections assigned to the STOL(St4)/tuf-d CaPsol genotype. Transmission of CaPsol was confirmed in replicate Wh7-25, which was inoculated exclusive with *R. quinquecostatus*. Although replicate Wh6–25 contained also *R. panzeri* and *R. cuspidatus* individuals, molecular screening showed that these specimens were CaPsol-negative, and therefore the infection could likely be attributed to *R. quinquecostatus* (**Figure 5A**; **Table 2**).

Semi-field cage experiment on sunflower confirmed high CaPsol transmission efficiency, with 23/25 plants testing positive by qPCR. Of these, 21 were additionally confirmed by nested PCR (*stamp* and *tuf*) using combined DNA dilutions of 1:30 (5 plants) and 1:100 (16 additional plants), resulting in 91% agreement between detection methods. The remaining two samples tested negative by 16S rRNA nested PCR. All infected plants carried the STOL(St4)/tuf-d genotype (**Figure 5A**). Because post-experimental identification of the introduced planthoppers was not feasible, species identity within the cage could not be verified beyond the initial identification of the source population as *R. quinquecostatus*. However, based on parallel transmission trials (**Table 2**), in which non-*R. quinquecostatus* species represented 8% of all individuals (22/272), non-target planthoppers were likely also present in the cage experiment.

Field-collected sunflower and wheat plants exhibited no CaPsol-associated symptoms. Following experimental inoculation, individual sunflower plants began to decline approximately after eight weeks and were sampled from roots prior to complete collapse (≤12 weeks). Wheat plants wilted shortly after transplantation into larger pots and were sampled within eight weeks. Most sugar beet plants declined after approximately 10 weeks, and root tissues were collected beforehand. Periwinkle plants developed characteristic symptoms, including leaf yellowing and phyllody, and declined approximately five months post-inoculation. In contrast, sunflower plants maintained under semi-field cage conditions remained asymptomatic in both inoculated and negative control treatments and were visually indistinguishable from surrounding field plants.

### 3.5 Contribution of *H. obsoletus* to sunflower-associated CaPsol epidemiology

Screening of the *H. obsoletus* population revealed a CaPsol infection rate of 46% (11/24). Genotyping of 10 strains identified five genetic variants: STOL(St4)/tuf-d (3), STOL(St4)/tuf-b (1), Rqg50(St1)/tuf-b (3), Rqg31(St2)/tuf-b (2), and GGY(St5)/tuf-b (1) (**Figure 5B**).

A high CaPsol incidence was detected in *C. arvensis* (90%; 18/20), with all genotyped strains belonging to the tuf-b type, predominantly Rqg31(St2) (9/10), with Rqg50(St1) detected in a single sample (**Figure 5B**).

In field-collected sunflower plants, CaPsol incidence was 60% (18/30) based on qPCR assays. As observed previously, discrepancies between detection methods were also evident in these samples. Nested PCR (*stamp* and *tuf*), performed using 1:100 diluted DNA, confirmed infection in 12/18 qPCR positive samples, corresponding to 67% agreement between methods. The remaining samples were negative by 16S rRNA nested PCR. All genotyped strains (10/10) belonged to the STOL(St4)/tuf-d genotype.

Transmission by *H. obsoletus* was confirmed in all experimental plant species, with CaPsol detected in all sunflower (8/8), sugar beet (6/6), and periwinkle replicates (2/2) (**Table 3**; **Figure 5B**). In sunflower, the STOL(St4)/tuf-d genotype predominated (7/8), while a single replicate carried Rqg50(St1)/tuf-b. In sugar beet, tuf-b genotypes prevailed, including Rqg31(St2)/tuf-b (3/6) and M5(St28)/tuf-b (1/6), whereas STOL(St4)/tuf-d was identified in two replicates. One sugar beet replicate (Sb5-25) was unintentionally exposed to two *R. quinquecostatus* individuals, one of which tested positive for CaPsol (**Table 3**). Periwinkle plants harbored Rqg31(St2)/tuf-b and M5(St28)/tuf-b genotypes.

**Table 3:** CaPsol-positive experimental plants obtained in transmission trials (2025) with *H. obsoletus*.

Experimental plant; CaPsol-positive/total replicates	Replicate ID	Planthoppers in transmission trial CaPsol-positive/total individuals^*^				CaPsol genotype *stamp*/*tuf*
		♂ Ho	♀ Ho	♂ Rq	♀ Rq	
Sunflower 8/8	Sf7-25	16	15	/	/	STOL(St4)/tuf-d
	Sf8-25	16	15	/	/	STOL(St4)/tuf-d
	Sf9-25	15	15	/	/	Rqg50(St1)/tuf-b
	Sf10-25	11	14	/	/	STOL(St4)/tuf-d
	SF11-25	23	10	/	/	STOL(St4)/tuf-d
	Sf12-25	12	13	/	/	STOL(St4)/tuf-d
	Sf13-25	18	17	/	/	STOL(St4)/tuf-d
	Sf14-25	24	9	/	/	STOL(St4)/tuf-d
Sugar beet 6/6	**Sb5-25**	20	16	1/1	0/1	Rqg31(St2)/tuf-b
	Sb7-25	24	12	/	/	Rqg31(St2)/tuf-b
	Sb8-25	13	16	/	/	Rqg31(St2)/tuf-b
	Sb9-25	24	13	/	/	STOL(St4)/tuf-d
	Sb10-25	23	20	/	/	M5(St28)/tuf-b
	Sb11-25	13	15	/	/	STOL(St4)/tuf-d
Periwinkle 2/2	W8-25	12	4	/	/	Rqg31(St2)/tuf-b
	W9-25	14	15	/	/	M5(St28)/tuf-b

The abbreviations used for planthopper species are: Ho (*H. obsoletus*) and Rq (*R. quinquecostatus*); sex symbols indicate the number of male and female specimens present in each replicate. Replicates shown in bold indicate the presence of CaPsol-positive non-target planthoppers during inoculation. (*) CaPsol infection is shown only for non-target planthopper individuals per replicate. Corresponding reference accession numbers for the identified genotypes: Rqg50 (St1; KC703019), Rqg31 (St2; KC703017), STOL (St4; FN813261), and M5 (St28; KP337316).

Symptom expression in experimentally inoculated plants was consistent with that observed in the *R. quinquecostatus* transmission experiments.

## 4 Discussion

Changes in crop rotation are increasingly being explored as a strategy to mitigate vector-borne disease pressure (Bressan, 2009; Pfitzer et al., 2024), yet their effectiveness against CaPsol remains unclear due to limited understanding of vector-crop interactions. Our results indicate that the sunflower-wheat sequence may facilitate, rather than disrupt, CaPsol persistence by hosting infected vector populations, with sunflower acting as an important epidemiological link. Shared nymphal habitats among multiple vector species create overlapping transmission pathways and increase epidemiological complexity. The consistent presence of *R. quinquecostatus* within both sunflower-wheat and maize-wheat sequences provides a plausible explanation for the origin of its populations colonizing different crops. Together with adult monitoring and emergence-cage data, these findings further support its co-occurrence with *R. panzeri* (Jović et al., 2009; Cvrković et al., 2014; Mitrović et al., 2016; Kosovac et al., 2023a). The capacity of *R. quinquecostatus* to dominate in below-ground communities in the maize-wheat sequence, alongside the presence of *R. panzeri* in the sunflower-wheat sequence, suggests that both vectors may contribute to cross-crop transmission beyond the studied rotation system.

Nymphs of *R. quinquecostatus* were consistently detected across both crop sequences, whereas other planthopper species showed temporal variability. Although *R. panzeri* nymphs were not detected in 2024, this likely reflects spatial aggregation of oviposition sites and nymphal populations, as adult monitoring confirmed the continued presence of the species. Despite the occurrence of *R. cuspidatus* in ruderal habitats adjacent to fields (Kosovac et al., 2023a; confirmed in this study, data not shown), it was rarely detected on sunflower and absent from emergence cages, providing limited evidence supporting an association with crops. Detection of *H. obsoletus* nymphs in both crop sequences, together with adult emergence from wheat following sunflower, was most likely linked to *C. arvensis*. While development of *H. obsoletus* on cultivated aromatic plants has been documented (Boudon-Padieu and Cousin, 1999; Chuche et al., 2018; Sémétey et al., 2018), its associations with arable crops remain unknown. More broadly, the ability of planthoppers to exploit multiple crops within rotation systems, as shown for *P. leporinus* nymphs across potato, sugar beet, and carrot, together with adult dispersal (Behrmann et al., 2023; Therhaag et al., 2024; Witczak et al., 2025), highlights adaptive flexibility and suggests functional connectivity between crops that may also apply to *Reptalus* spp.

To our knowledge, this study provides the first evidence identifying sunflower as a developmental host for planthoppers. While wheat has previously been reported as an overwintering host for *R. panzeri* (Jović et al., 2009), our findings indicate that it can be similarly utilized by *R. quinquecostatus*. The prevalence of fourth-instar nymphs (N4) on sunflower roots during autumn suggests overwintering at this stage, whereas the transition to N5 on wheat in late spring indicates a narrow interval between the final molt and adult emergence. Vertical soil movement was also observed, with nymphs occupying upper soil layers during autumn, shifting deeper in early spring, and returning toward the surface by May-June. This pattern is consistent with temperature-driven movement and possible diapause behavior in planthoppers (Bressan et al., 2009; Behrmann et al., 2022). While the standardized sampling depth may have underestimated nymphal abundance during early spring, it successfully documented the completion of subterranean development, as the detection of N5 nymphs in late spring together with adult emergence in cages confirmed the role of the assessed crop sequences in completing the vector life cycle under field conditions.

The absence of CaPsol in field-collected wheat samples, together with low transmission efficiency and previously reported low infection rates (Jović et al., 2009), indicates that wheat primarily supports vector development rather than acting as an important CaPsol reservoir. In contrast, the 100% infection rate observed in field-collected sunflower roots in 2025, together with high infection levels (up to 83%) in sunflower-associated N4 *R. quinquecostatus* nymphs, suggests that sunflower may function as a source of pathogen acquisition. These findings support an epidemiological cycle in which *R. quinquecostatus* likely acquires CaPsol from sunflower roots, overwinters on wheat, and emerges as an infective adult. Although confirmation of this pathway using laboratory-reared vector population is still required, present study provides a strong foundation for future experimental validation. High transmission efficiency to sunflower, together with successful transmission in replicates involving CaPsol-infected *R. panzeri* and in trials using *H. obsoletus* populations, suggests that multiple vector species may contribute to the seasonal re-establishment of sunflower as a pathogen reservoir and facilitate cross-crop spread of CaPsol.

Post-experimental verification of insect identity represents an critical methodological step given the cryptic co-occurrence of vectors, as *R. quinquecostatus* and *R. panzeri* are morphologically indistinguishable during field sampling. In Kosovac et al. (2023a, b), CaPsol transmission by *R. quinquecostatus* was evaluated using individually exposed plants, whereas Duduk et al. (2023a) inferred species identity from a subset of specimens prior to release into cages, without post-experimental verification. Such an approach may not ensure taxonomic homogeneity, a limitation encountered in this study. In both experimental years, initial screening of *Reptalus* populations collected from wheat following sunflower suggested the exclusive presence of *R. quinquecostatus*. However, post-trial verification revealed the presence of non-target vector species. In 2024, in 1/22 replicates *R. panzeri* individuals were enrolled in inoculations, whereas in 2025 non-target vectors were identified in 7/16 CaPsol-infected replicates, substantially complicating and compromising interpretation of the transmission results.

Species composition of planthoppers at the IFVCNS experimental fields varied among crops and years, likely reflecting local microecological conditions, including crop diversity and rotation scheme. This variability warrants caution when extrapolating findings to other agroecosystems. Consistent with this, adult planthopper monitoring data from Slovakia showed that *R. panzeri* dominated over *R. quinquecostatus* in RTD-affected sugar beet fields in 2025, accounting for 52-88% of individuals across most of monitored sites (United Beet Seeds B.V. (UBS), internal data). Although *R. panzeri* was reported to transmit CaPsol to grapevine and potato (Cvrković et al., 2014; Mitrović et al., 2016), its role within multi-crop systems remains difficult to isolate experimentally because it co-occurs with *R. quinquecostatus*. Our findings suggest that *R. panzeri* may utilize sunflower, in addition to maize, as a developmental host and pathogen reservoir for nymphs before overwintering in the following wheat crop. Such multi-crop life cycles of both *R. quinquecostatus* and *R. panzeri* likely facilitate dispersal of infective adults and contribute to CaPsol spread to non-host crops.

A discrepancy between qPCR and nested PCR detection was observed in sunflower samples, consistent with Witczak et al. (2025), who also reported higher detection sensitivity of qPCR, indicating that negative results do not imply absence of infection. Reduced nested PCR sensitivity in sunflower may result from secondary metabolites, including polyphenols and polysaccharides, that interfere with the amplification efficiency, together with the higher sensitivity of qPCR (Schrader et al., 2012). Increased template dilution improved nested PCR detection, while longer end-point amplicons likely increased the risk of false negatives in low-titer samples. Notably, CaPsol was not detected in sunflower leaf samples collected in 2024 using either qPCR or nested PCR, despite testing different template dilutions, whereas root samples collected in 2025 showed high infection rates. This pattern suggests an uneven distribution of CaPsol within sunflower tissues, with pathogen accumulation potentially concentrated in roots at detectable levels despite remaining undetected in aerial tissues. Tissue-specific physiological or defense-related responses may additionally influence phytoplasma colonization, persistence, and accumulation within different plant organs (Dermastia, 2019; Landi et al., 2019).

Previous studies reported predominance of the STOL(St4)/tuf-d CaPsol genotype in Pannonian sugar beet RTD outbreaks, suggesting an association with a broader epidemiological cycle rather than a transmission pathway exclusively linked to this crop (Ćurčić et al., 2021b). Our findings, combining high CaPsol infection rates with genotyping across field and experimental samples, confirm the prevalence of the STOL(St4)/tuf-d genotype at the IFVCNS site. Although natural vegetation may serve as a reservoir for this genotype (Cvrković et al., 2022), and the role of *Prunus* and *Crataegus* plants as feeding hosts for adult *R. quinquecostatus* remains unclear (Kosovac et al., 2023b), these habitats likely represent a background reservoir. In contrast, our findings suggest that CaPsol outbreaks mediated by *Reptalus* spp. are likely driven primarily by crop-associated transmission cycles facilitated by established rotation systems. Notably, the same genotype also predominated in transmission experiments involving *H. obsoletus*, despite not being detected in *C. arvensis* samples in the present study. However, this weedy host was previously shown to harbor this genotype at the same site (Kosovac et al., 2023a).

In Serbia, sunflower is commonly cultivated in cereal-based rotations following maize or wheat (Crnobarac et al., 2016). However, from the perspective of vector life cycle dynamics and CaPsol epidemiology, the subsequent crop represents a more critical focal point than the preceding one. Under regional production conditions, sunflower yield is generally not affected by rotation differences, and together with the asymptomatic nature of infection, this suggests that its epidemiological role and risk to subsequent crops may remain unnoticed under standard agronomic practices. Maize is predominantly cultivated in two-crop rotations in Serbia with wheat (60%) or soybean (15%), as well as in continuous cropping (15%) (Videnović et al., 2013). In maize-dominated regions, this crop should be regarded not only as a symptomatic CaPsol host but also as an important crop supporting *Reptalus* spp. populations and facilitating pathogen spread.

Sugar beet occupies only 2-3% of arable land in Serbia and is typically incorporated into cereal-based rotation systems (Bojović et al., 2022; Živaljević et al., 2024). Consequently, it likely plays a relatively minor role in regional crop connectivity compared with the dominant sequences structured by sunflower, maize, and wheat. Although sugar beet is affected by CaPsol, our findings do not permit a definitive assessment of its broader epidemiological role. Specifically, it remains unclear whether this crop acts as a transient CaPsol spillover host exposed to infective vectors originating from neighboring crops, or if it can sustain *Reptalus* spp. populations under certain crop rotations. The absence of nymphs on sugar beet roots at assessed experimental site suggests that this crop may be an unsuitable host for oviposition. However, these observations require further validation across diverse agroecological regions and different crop sequences.

The presence of *R. quinquecostatus* adults in oat following sunflower at the same site (Popov et al., 2024) suggests that overwintering may also extend to other cereal hosts. A comparable pattern has been reported for *P. leporinus*, in which barley supports nymphal development, although at substantially lower levels, leading to its consideration in vector management strategies (Bressan, 2009). However, the identification of major production crops as hosts for both CaPsol and multiple vector species presents a considerable management challenge. Because these crops are economically indispensable throughout Europe, implementation of alternative crop rotations aimed at disrupting vector life cycle remains both logistically and economically difficult.

The current taxonomic instability surrounding *R. quinquecostatus* sensu Holzinger et al. (2003) extends beyond formal nomenclature, representing a significant source of uncertainty in applied research. A comprehensive entomological and taxonomic approach is therefore essential to ensure the validity of vector-host associations and to avoid potential discrepancies in epidemiological interpretation and disease management strategies arising from unresolved taxonomy and inaccurate species identification (Paredes-Esquivel et al., 2009; Kosovac et al., 2018; Lachish and Murray, 2018; Stejskal et al., 2025). Given these complexities, we retain the name *R. quinquecostatus* in the future research to ensure the consistency and traceability of our data until a comprehensive taxonomic revision provides further consensus.

A comprehensive understanding of CaPsol-associated diseases requires an integrated approach that considers vector biology and phytopathological aspects as intrinsic components of the same pathosystem. By linking the life cycle phenology of *R. quinquecostatus* and *R. panzeri*, this study provides a framework for understanding multi-vector stolbur disease dynamics. Our findings highlight the importance of rigorous species identity verification for both planthopper nymphs and adults using integrated morphological and molecular diagnostics. Efficient CaPsol monitoring requires not only sensitive molecular detection incorporating complementary qPCR and nested PCR approaches, but also consideration of asymptomatic reservoirs within the broader agroecosystem. Future research should focus on validating the epidemiological role of sunflower and monitoring vector development across production-scale conditions. While substituting key production crops may be challenging, crop rotation-based strategies that disrupt vector development remain a promising approach for reducing the impact and spread of CaPsol across European agroecosystems.

## Data Availability

The datasets presented in this study can be found in online repositories. The names of the repository/repositories and accession number(s) can be found below: https://www.ncbi.nlm.nih.gov/genbank/, PZ289166–PZ289193.
